# Next generation *in vitro* liver model design: Combining a permeable polystyrene membrane with a transdifferentiated cell line

**DOI:** 10.1016/j.memsci.2018.07.063

**Published:** 2018-11-01

**Authors:** Kim A. Luetchford, Nelly Wung, Iain S. Argyle, Michael P. Storm, Stephen D. Weston, David Tosh, Marianne J. Ellis

**Affiliations:** a Department of Chemical Engineering, University of Bath, Bath BA2 7AY, UK; b Centre for Regenerative Medicine, Department of Biology and Biochemistry, University of Bath, Bath BA2 7AY, UK

**Keywords:** Hollow fibre membranes, Polystyrene, Porogen, Transdifferentiation, *In vitro* liver models

## Abstract

Herein we describe the manufacture and characterisation of biocompatible, porous polystyrene membranes, suitable for cell culture. Though widely used in traditional cell culture, polystyrene has not been used as a hollow fibre membrane due to its hydrophobicity and non-porous structure.

Here, we use microcrystalline sodium chloride (4.7 ± 1.3 µm) to control the porosity of polystyrene membranes and oxygen plasma surface treatment to reduce hydrophobicity. Increased porogen concentration correlates to increased surface pore density, macrovoid formation, gas permeability and mean pore size, but a decrease in mechanical strength. For tissue engineering applications, membranes spun from casting solutions containing 40% (w/w) sodium chloride represent a compromise between strength and permeability, having surface pore density of 208.2 ± 29.7 pores/mm^2^, mean surface pore size of 2.3 ± 0.7 µm, and Young's modulus of 115.0 ± 8.2 MPa.

We demonstrate the biocompatibility of the material with an exciting cell line-media combination: transdifferentiation of the AR42J-B13 pancreatic cell line to hepatocyte-like cells. Treatment of AR42J-B13 with dexamethasone/oncostatin-M over 14 days induces transdifferentiation towards a hepatic phenotype. There was a distinct loss of the pancreatic phenotype, shown through loss of expression of the pancreatic marker amylase, and gain of the hepatic phenotype, shown through induction of expression of the hepatic markers transferrin, carbamoylphosphate synthetase and glutamine synthetase.

The combination of this membrane fabrication method and demonstration of biocompatibility of the transdifferentiated hepatocytes provides a novel, superior, alternative design for *in vitro* liver models and bioartificial liver devices.

## Highlights

• The conditions of membrane production are mild and economically viable.
• Microcrystalline sodium chloride enables controlled porosity of asymmetric membranes.
• Biocompatible non-biodegradable membranes support cell viability and transdifferentiation.
• Transdifferentiated AR42J-B13 cells offer potential for use in *in vitro* liver models.

## Graphical abstract

**Figure:**
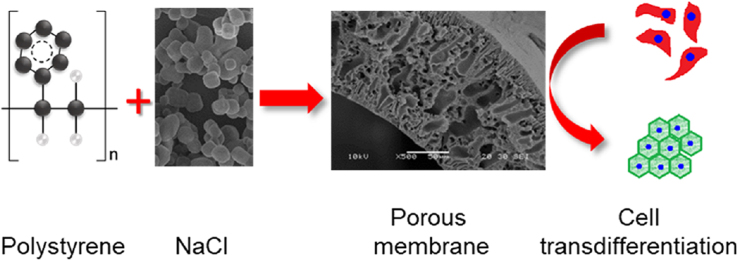

## 1 Introduction

The use of membranes as cell scaffolds is of key interest in the development of *in vitro* drug screening assays. Cells cultured in membrane bioreactors experience a more *in vivo-*like environment than those in traditional two-dimensional cell culture [1]. Culturing cells under physiologically relevant conditions can create more realistic and accurate metabolic responses to drug testing [2], [3]. By culturing cells on one side of a membrane, with bulk fluid flow on the opposite side, mass transfer rates become independent of the shear forces experienced by the cells [4]. At the same time, fluid flow simultaneously allows for a constant and uniform supply of fresh media to the cells and offers efficient removal of waste metabolites and other extracellular products. The use of membranes in hollow fibre bioreactors (HFBs) also allows for simulation of specific organ functions: for example, human liver and kidney HFB models have been demonstrated [5], [6].

To exploit HFBs for cell culture applications, there is an intrinsic dependence on the consistency and quality of the membrane scaffolds themselves. Given the large number of possible bioartificial models which could be recreated in HFBs (theoretically, any vascularised tissue), reliance on a commercially available supply of hollow fibres does not currently offer the degree of refinement required in terms of material stiffness, pore size, porosity and surface chemical properties.

Both biodegradable and non-biodegradable polymers have distinct and complementary properties when used as tissue culture scaffolds. In regenerative medicine, biodegradable scaffolds allow for the culture of cells as the eventual degradation of the scaffold into non-toxic constituents leaves behind the tissue engineered construct. However, non-biodegradable polymers are more appropriate for long-term cell expansion and bioartificial organs, where constant environmental support for an indeterminate amount of time is key. Traditionally, adherent tissue culture flasks are made from polystyrene, a durable, inexpensive non-biodegradable polymer that is established as a mechanically stable and biocompatible scaffold material [7], [8], [9]. However, other polystyrene substrates are used extremely rarely, probably as a result of their hydrophobic and non-porous nature.

There are limited examples of porous polystyrene membranes reported in the literature and to the best of the authors’ knowledge, no reports of polystyrene hollow fibre membranes suitable for cell culture at all. The existing reports either require the use of high pressures and supercritical fluids for membrane fabrication, or produce membranes with dimensions unsuitable for cell culture [10], [11].

Membrane production by phase inversion relies on polymer precipitation upon immersion in a non-solvent. The consequent de-mixing of the solvent-polymer dispersion into the non-solvent creates the characteristic porous network of a polymeric membrane [12]. To enhance the formation of pores, incorporation of pore-forming agents (porogens) into the solvent-polymer solution is a well-documented strategy [13]. The chosen porogen normally has limited solubility in the solvent-polymer solution, but is readily soluble in the non-solvent, enabling removal by dissolution upon phase inversion. Typical porogens are inert and readily soluble in water, and include polyvinylpyrrolidone (PVP) and polyethylene glycols (PEGs). Salt crystals are often used as porogens in tissue engineering scaffolds, most typically when the aim is to create large pores, in the order of 200 µm, to enable cells to infiltrate and migrate into the scaffolds [14], [15], [16], [17]. The previously successful use of sodium chloride as a porogen in tissue engineering gives confidence in its use in our application – the production of a microporous polystyrene membrane using a salt porogen. Here we produce microcrystalline sodium chloride, using a method developed by Marshall [18], which to date has not been used as a porogen for polystyrene. We describe the manufacture of polystyrene flat sheet and hollow fibre membranes and analyse their physical properties.

To demonstrate biocompatibility of the resulting membranes, we compare the viability and transdifferentiation potential of the pancreatic AR42J-B13 (B13) cell line on flat sheet porous polystyrene membranes, flat sheet non-porous polystyrene membranes, and traditional tissue culture polystyrene (TCPS). B13 cells can be induced to convert from pancreatic to hepatocyte-like cells (HLCs) following culture with the synthetic glucocorticoid dexamethasone (Dex). The phenotype of the cells can be enhanced by co-culture with Dex and oncostatin M (OSM) [19]. The use of transdifferentiated B13 cells as a liver model has advantages over using hepatoma cancer cell lines or primary hepatocytes. Primary hepatocytes are difficult to obtain, cannot be expanded *in vitro*, and dedifferentiate rapidly in suspension [20]. Meanwhile, hepatoma cell lines such as HepG2 cells can be sub-cultured successfully but show extremely low drug metabolism activity [21]. However, B13 cells readily proliferate *in vitro*, and following transdifferentiation into HLCs they function at a level similar to freshly isolated rat hepatocytes [22]. Recently published work also suggests B13 culture can be adapted to serum-free conditions, removing barriers to their clinical use [23].

In this work we describe the culture of transdifferentiated B13 cells on our novel porous polystyrene membranes – a combination likely to help generate better *in vitro* liver models by creating more *in vivo*-like culture environments using physiologically relevant cells at high densities.

## 2 Experimental

### 2.1 Microcrystalline sodium chloride production

Sodium chloride crystals were prepared as detailed by Marshall [18]. Briefly, a saturated solution of sodium chloride (Sigma-Aldrich) was prepared and a 5% additional volume of reverse osmosis (RO) water was added. Four 25 mL aliquots of this solution were frozen in dry ice, then broken apart and shaken vigorously in 2 L pure ethanol at − 20 °C (Sigma-Aldrich). Once the frozen salt was completely melted, the precipitate was collected by vacuum filtration, prior to lyophilisation. This product is referred to in this paper as microcrystalline sodium chloride.

For comparison, dry sodium chloride crystals (Sigma-Aldrich) were thoroughly ground in a pestle and mortar.

Sodium chloride crystal face lengths were assessed by analysing light micrographs, obtained using an IX51 microscope (Olympus). Face lengths were measured using Cell^P software (Olympus).

### 2.2 Formulation of membrane casting solutions

Casting solutions were formulated using various mass ratios of polystyrene and microcrystalline sodium chloride, as detailed in Table 1. The casting solutions were prepared by first dispersing the appropriate mass of microcrystalline sodium chloride crystals in 20 g (19.42 mL) of n-methyl-2-pyrrolidone (NMP; Sigma-Aldrich). 5 g of polystyrene (average molecular weight ~ 280,000, Sigma-Aldrich) was then added to the mixture and agitated until fully incorporated.

**Table 1:** Polymer-salt-solvent formulations.

**Nomenclature**	**Mass of salt (g)**	**Mass of NMP (g)**	**Mass of polystyrene (g)**	**Polystyrene to salt ratio**	**Mass of salt as a percentage of mass of polystyrene (%)**
PX0	0	20	5.0	1:0	0
PX10	0.5	20	5.0	9:1	10
PX20	1.0	20	5.0	4:1	20
PX40	2.0	20	5.0	3:2	40
PX60	3.0	20	5.0	2:3	60

### 2.3 Flat sheet and hollow fibre membrane preparation

Flat sheet membranes were produced by immersion precipitation, using RO water as the non-solvent. Casting solutions were spread on 100 × 200 mm glass panes using rollers, then fully immersed in RO water and left to soak to allow for membrane precipitation. The spacing between roller and glass was fixed using 340 µm wire (28 AWG). The water was changed twice a day for 3 days, for solvent and salt removal. The membranes were air-dried and stored in a desiccator prior to use.

Hollow fibres were prepared by a wet spinning technique detailed elsewhere [3]. The casting solution-containing tank was well mixed prior to spinning to ensure uniform salt dispersion. RO water was used as the non-solvent, and the resultant fibres underwent the same washing and drying regime as the flat sheet membranes.

To hydrophilise the membranes for cell culture applications, samples were exposed to oxygen plasma under a vacuum. Samples were placed in a capacitively-coupled plasma chamber (Zepto-Diener) and treated with oxygen plasma at a power of 25 W for 30 s with a flow rate of approximately 40 cm^3^/minute.

### 2.4 AR42J-B13 cell culture and transdifferentiation to hepatocyte-like cells

Rat pancreatic AR42J-B13 (B13) cells are a sub-clone of the parent line AR42J (provided by Itaru Kojima, Japan). Cells were maintained in standard culture conditions, in complete medium consisting of Dulbecco's Minimum Essential medium (DMEM; D5546, Sigma-Aldrich), 10% (v/v) foetal bovine serum (Gibco), 1% (v/v) L-glutamine (Sigma-Aldrich) and 1% (v/v) penicillin-streptomycin (Sigma-Aldrich) as previously described [19]. Medium was changed every 2 days thereafter.

For transdifferentiation to hepatocyte-like cells (HLCs), B13 cells were seeded at a density of 10,000 cells/cm^2^ and initially cultured under maintenance conditions for 24 h after seeding. After this time period, to induce transdifferentiation the maintenance culture medium was additionally supplemented for 14 days with 1 μM dexamethasone (Dex) (Sigma-Aldrich) and 10 ng/mL Oncostatin-M (OSM) (PeproTech). The supplemented medium was replaced every 2 days.

### 2.5 Bioreactor preparation

A custom flat sheet membrane bioreactor was used to fix oxygen plasma treated membranes into place for cell culture applications. The bioreactor was designed to clamp membranes between two plates: one solid and one perforated with wells to allow for cell culture on the membrane surface. Construction of the flat sheet membrane bioreactor was performed in a Class II safety cabinet and the module components and silicone gaskets were sterilised by autoclaving prior to assembly.

PX membranes, initially treated with 70% (v/v) ethanol (Sigma-Aldrich), were sealed into the custom 24-well plate polycarbonate modules, between silicone gaskets, leaving a membrane surface area of 1.9 cm^2^ per well exposed for cell culture (comparable to commercial well plate dimensions). Membranes were allowed to fully dry, and were then sterilised by immersion in 1% (v/v) antibiotic-antimycotic solution (Sigma-Aldrich) in phosphate buffered saline (PBS) at 4 °C for 24 h, and were subsequently rinsed 3 times in PBS prior to cell seeding [24].

### 2.6 Membrane characterisation

#### 2.6.1 Mechanical property analysis

In order to gauge the effect that microcrystalline sodium chloride had on membrane mechanical integrity, flat sheet membranes were destructively tested on an Instron 5965 universal testing machine fitted with a 1 kN load cell. Dumbbell-shaped sections of the membranes were cut with a width of 4 mm and a narrow section length of 30 mm. The thickness of each sample was measured using a micrometer (Mitutoyo). The wider ends of the dumbbell shapes were clamped, and extension tests were performed at a rate of 0.5 mm s^−1^ until sample failure.

Ultimate tensile strength was calculated by dividing the maximum force at break by the sample cross-sectional area. Extension at break was gauged by finding the difference between the zero point and the distance moved at sample failure. The apparent Young's modulus was calculated from the gradient of the stress-strain curve.

#### 2.6.2 Contact angle measurement

Contact angles of flat sheet membranes were measured on an OCA15 goniometer (DataPhysics). 1 µL of RO water was placed on the surface of each membrane at room temperature. Contact angles were measured using integrated droplet image detection software and calculated based on the Laplace-Young equation.

#### 2.6.3 Thermogravimetric analysis

Approximately 10 mg of dry PX sample was placed in an open crucible in a TGA instrument (Setaram Setsys Evolution 16/18), which was heated to 700 °C in an atmosphere of dry air, at a flow rate of 20 mL/minute. The heating rate was 10 K/minute. The mass signal was corrected to remove the contribution of buoyancy effect, by subtracting the data from an identical run with the sample holder left empty. Two replicates were analysed for each membrane.

#### 2.6.4 Scanning electron microscopy

Scanning electron microscopy (SEM) was performed on membrane samples and sodium chloride samples. Skin layer top-down membrane samples and sodium chloride samples were prepared by lyophilising and gold sputter-coating (Edwards Sputter Coater 5150B). Membrane cross-sections were prepared by immersing the samples in liquid nitrogen, fracturing across the structure, and lyophilising, followed by gold sputter-coating. All samples were imaged at 10 kV using an SEM 6480LV microscope (Jeol).

#### 2.6.5 Pore size distribution and surface porosity

SEM images of membrane surfaces were used to gauge size distributions of surface pores. Images were converted to binary and analysed using ImageJ software (NIH). Measurements of area, perimeter and Feret's diameter were used to calculate circularity of the pores. The product of circularity and Feret's diameter was used to give geometric pore diameters [25]. Histograms were produced from data and fitted using a 3 parameter Gaussian distribution. For each membrane formulation, measurements were taken from 8 representative images (each 0.13 mm^2^) from 4 separate membranes.

#### 2.6.6 Hollow fibre permeability

Hollow fibre permeability was tested using nitrogen gas permeation at room temperature. Hollow fibre membranes were fixed into stainless steel modules by sealing the extracapillary space between the fibres and module walls at the inlet and sealing the fibre lumens at the outlet. This enabled a flow of gas into the lumen. Transmembrane pressure (TMP) was increased from atmospheric levels up to approximately 1.0 bar. Gas flow rates into the module were measured using a rotameter. Measurements were recorded at arbitrary TMPs for at least 4 comparable modules. Mean pore size was calculated using a previously developed method and correlation [26].

The permeability of PX0 and PX40 hollow fibre membranes was also tested using water permeation at room temperature. For each experiment, a bundle of three fibres was fixed inside a glass module which allowed for two directions of outflow: through the fibre lumen (retentate flow) and through the fibre walls (permeate flow). A clamp was placed over the retentate line, and the permeate flow output was measured by mass. The fibres were pre-wetted with 70% ethanol, which was then washed out with distilled water before recording data. The system was filled with distilled water and a degree of permeate flow was induced, to ensure the system was stabilised prior to recording data. Distilled water was pumped through the system as the clamp was closed in increments, and pressure and permeation were recorded.

### 2.7 Analysis of biocompatibility

#### 2.7.1 Cell viability

Cell viability was visualised using the LIVE/DEAD Viability/Cytotoxicity Kit (Invitrogen). Cells were seeded at a density of 20,000 cells/cm^2^ then incubated for 48 h at 37 °C in 5% CO_2_. After incubation, the cells were washed gently in PBS before adding 1 μM calcein AM and 1 μM ethidium homodimer-1 in PBS, and incubated for 30 min. Fluorescence was visualised on an inverted microscope. The number of cells fluorescent with either calcein AM (green) or ethidium homodimer-1 (red) was counted for 6 independent fields of view (FOV) per replicate and normalised against the total number of cells in the FOV. A mean and standard error of percentage live and dead cells for each culture substrate were calculated from 3 independent experiments.

#### 2.7.2 Immunofluorescent staining of cell cultures

B13 cells were cultured as previously described in Section 2.4 and cultures were maintained under both standard and transdifferentiation conditions, for 4 or 14 days respectively. After this time, cells were immunostained as previously described [19]. Briefly, samples were washed in PBS and fixed with 4% paraformaldehyde in PBS for 20 min at room temperature. The cells were permeabilised with 0.1% (v/v) Triton X-100 in PBS for 20 min and blocked in 2% (v/v) blocking buffer (Roche) for 30 min before incubation with primary antibodies overnight at 4 °C, followed by secondary antibodies, and subsequent staining with 2-(4-Amidinophenyl)-6-indolecarbamidine dihydrochloride (DAPI) diluted 1:1000 in PBS.

Antibodies were diluted as follows: rabbit anti-amylase 1:100 (Sigma-Aldrich), mouse anti-glutamine synthetase (GS) 1:300 (BD Transduction Laboratories), rabbit anti-carbamoylphosphate synthetase-1 (CPS-1) 1:300 (a generous gift from Wouter Lamers) and rabbit anti-transferrin (TFN) 1:100 (Dako). Anti-mouse and anti-rabbit Alexa Fluor 488 conjugated antibodies (Vector Laboratories), and anti-rabbit Alexa Fluor 594 conjugated antibodies (Invitrogen) were used at a 1:500 dilution.

#### 2.7.3 Serum albumin secretion

B13 cells were cultured as previously described in Section 2.4. On day 13 of transdifferentiation treatment, the culture medium was changed to serum-free medium, supplemented with Dex and OSM. On day 14, the serum-free medium was removed from the cells and assayed for secreted albumin from the transdifferentiated HLCs, using a rat albumin enzyme-linked immunosorbent assay (ELISA; Bethyl Labs) according to the manufacturer's instructions.

Albumin secretion data was normalised by total protein. To quantify this, cells were washed with PBS, then lysed with RIPA Lysis and Extraction Buffer (Thermo Fisher Scientific) containing a 1:100 dilution of protease inhibitor cocktail. Total protein in the subsequent cell lysate was quantified using the Pierce BCA assay kit (Thermo Fisher Scientific).

### 2.8 Statistical analysis

Data is quoted as mean ± standard deviation (SD) unless otherwise stated. Statistical analysis was performed using one-way analysis of variance (ANOVA) with a post-hoc Holm-Sidak test, using SigmaPlot 12.3 (Systat Software Inc.), unless otherwise stated. A value of p < 0.05 was considered statistically significant.

## 3 Results

### 3.1 Flat sheet membrane characterisation

#### 3.1.1 Microcrystalline sodium chloride production

**Fig. 1:**
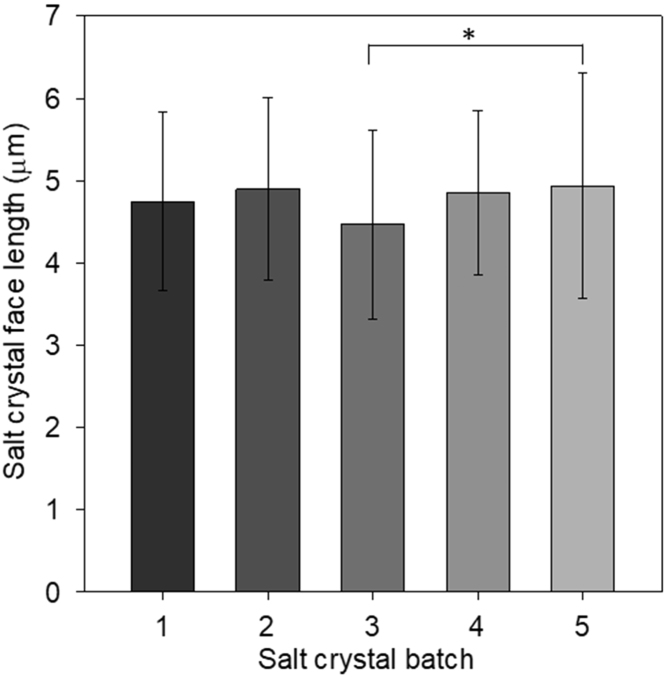
Size of microcrystalline sodium chloride produced in five separate batches. Data shown represents mean ± SD (n = 74–115). * indicates p < 0.05 for the indicated values.

Microcrystalline sodium chloride samples were prepared as described, and analysis of the size data (Fig. 1) shows the reproducibility of the method across different batches. The mean nominal face length in different batches ranged from 4.5 ± 1.2 µm to a maximum of 4.9 ± 1.4 µm (mean ± SD), with an overall mean of 4.7 ± 1.3 µm. A representative image of the type used for sizing the crystals is shown in Fig. S1a.

#### 3.1.2 Membrane composition

**Fig. 2:**
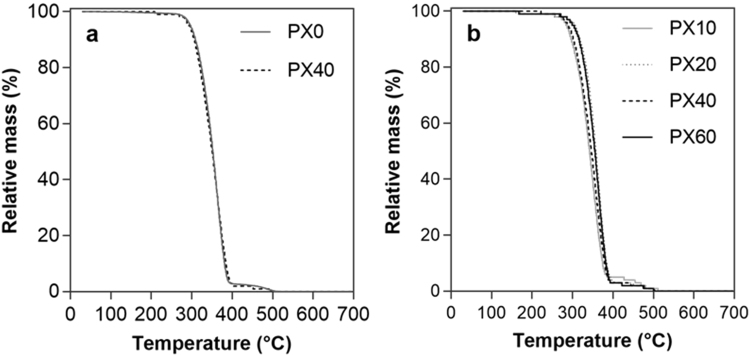
TGA curves of a) selected PX flat sheet membranes; and b) PX hollow fibre membranes.

To confirm the absence of salt in the final membrane products, the samples were analysed by TGA (Fig. 2). Between 300 °C and 400 °C, 95% of the mass of the membranes is lost, with the remaining 5% lost by 500 °C. This in line with literature reports suggesting polystyrene is entirely burnt by 500 °C [27] (whereas sodium chloride only melts above 800 °C [28]). The remaining mass of the samples is low and in the order of the detection limit of the equipment (5 µg). It is extremely unlikely any salt remains in the membranes. In addition, there is no difference between the TGA curves of PX0 and PX40 flat sheet membranes (Fig. 2a), nor between the TGA curves of HF membranes produced using different quantities of salt (Fig. 2b).

#### 3.1.3 Mechanical property analysis

**Fig. 3:**
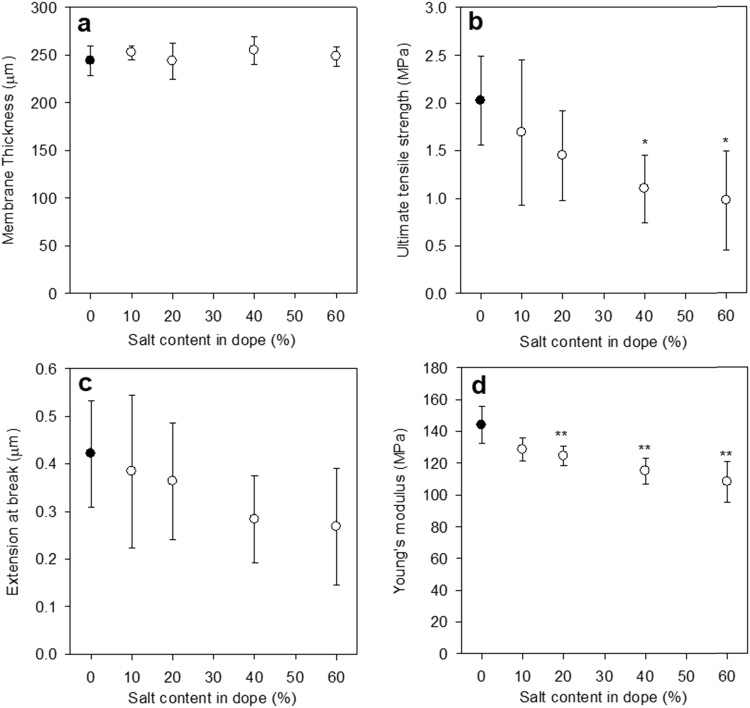
Properties of flat sheet membranes. (a) Thickness, (b) ultimate tensile strength, (c) extension at break and (d) Young's modulus. Data shown represents mean ± SD (n = 3–15). Asterisks indicate significance relative to the control of 0% salt content (* p < 0.05; ** p < 0.001).

The thicknesses of the membranes were measured as described in Section 2.6.1 (Fig. 3a). Membranes were of consistent thicknesses with a mean of 249 ± 20 µm. There was no significant difference in the thicknesses of membranes cast with different proportions of salt content. The membranes were cast at a thickness of 340 µm, indicating that a one-dimensional contraction of around 27% occurred during precipitation and drying.

Ultimate tensile strength was calculated for each membrane type (Fig. 3b). The strongest material was the membrane containing no salt (PX0) – this corresponds to the expectation that the membrane with no salt would be the least porous. Though the differences in ultimate tensile strength were only significant for PX40 and PX60 membranes with respect to the control PX0 (both p < 0.05), the data shows a trend of decreased strength with increased salt incorporation. ANOVA analysis revealed an overall trend significance of p < 0.001.

The extension at break (Fig. 3c) of the membranes also decreased with increased salt incorporation. The overall trend significance of p < 0.05 was lower than for the ultimate tensile strength, and discrete comparisons revealed no significance. The brittleness of the polymer was such that no sample yielding was apparent in the force curves generated.

Finally, Young's moduli were calculated for the membranes (Fig. 3d), based on the elastic region of the stress-strain curves. The Young's modulus values for PX20, PX40 and PX60 were all significantly lower than the PX0 value. Young's modulus appeared to decrease with increased salt concentration, suggesting the higher the salt concentration, the less stiff the resultant membrane. This again supports the assumption that increasing salt in the casting solution resulted in more porous membranes and hence decreased mechanical integrity.

#### 3.1.4 Membrane hydrophobicity

**Fig. 4:**
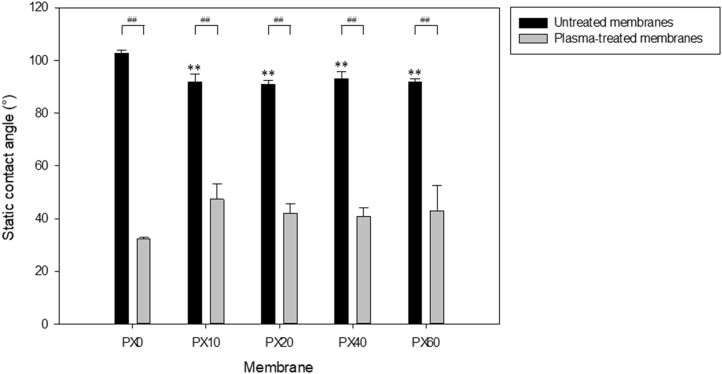
Contact angle of membranes with and without oxygen plasma treatment. Data shown represents mean ± SD (n = 3–14). ** indicates significance with respect to PX0 (p < 0.001); ## indicates p < 0.001 for the indicated values.

Membrane hydrophobicity, before and after oxygen plasma treatment, was determined from the contact angle measurements performed on flat sheet membranes (Fig. 4). The membranes cast from salt-containing solutions had significantly lower contact angles than the PX0 control membranes (p < 0.001) before oxygen plasma treatment. Following treatment, there was a significant reduction in the contact angles measured for all of the membranes, indicating a decrease in surface hydrophobicity.

#### 3.1.5 Morphological analysis

**Fig. 5:**
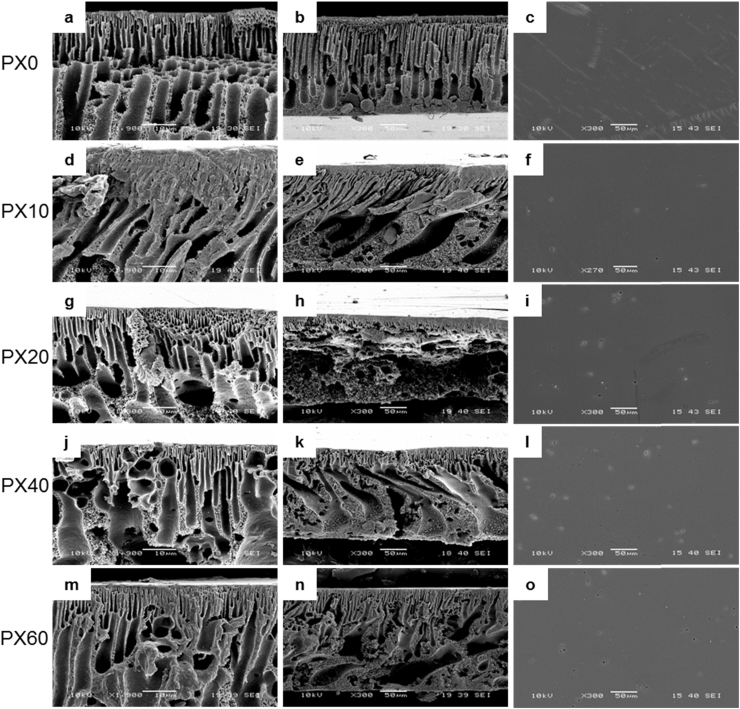
Representative SEM micrographs of flat sheet membrane cross-sections and surfaces. (a, b, c) PX0, (d, e, f) PX10, (g, h, i) PX20, (j, k, l) PX40 and (m, n, o) PX60. Scale bars of left hand column represent 10 µm; scale bars of middle and right represent 50 µm.

We determined the morphology of the flat sheet membranes by SEM (Fig. 5). For PX0, the structure shows the clear narrowing of pores from bottom to top (Fig. 5a) and a distinct thin top layer is apparent. This skin layer, when viewed from above (Fig. 5c), has no pores. The sub-structure shows some uniformity in macrovoid width, with the bottom most cavities having a maximum diameter of approximately 25 µm.

For the membranes cast from salt-containing solutions, there is more distinction between the top skin layer and the porous structure below. There are larger macrovoids in the sub-structure, and the skin layer has been narrowed as a result, resulting in pores on the skin surface. There is increased pore interconnectivity, with PX40 (Fig. 5j) showing particularly elongated pores spanning the whole membrane section, compared to the well-defined dual layer structure shown in PX0 (Fig. 5a).

**Fig. 6:**
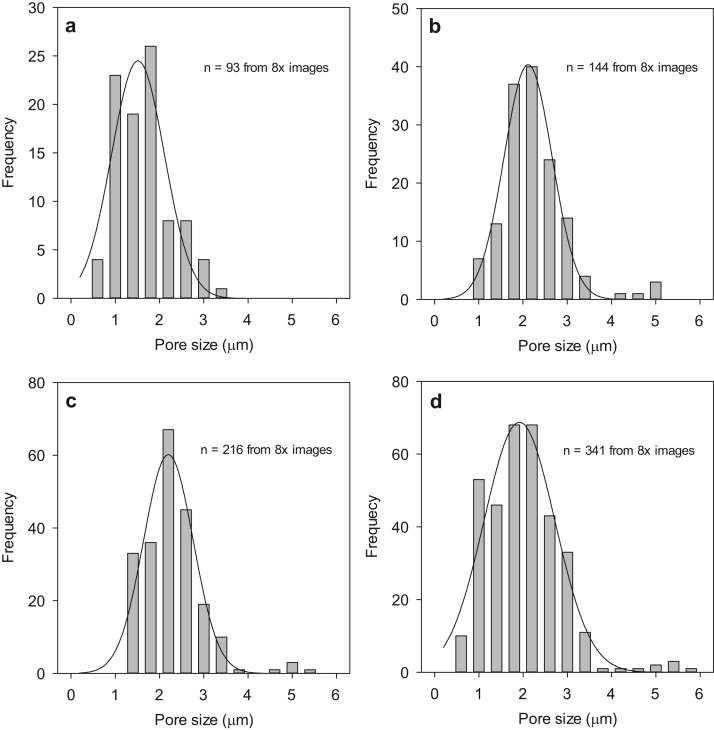
Surface pore size distribution histograms and respective Gaussian fits as deduced from image analysis for flat sheet membranes of (a) PX10, (b) PX20, (c) PX40, and (d) PX60. PX0 is not shown as no surface pores were detected.

Analysis of the top-down SEM images revealed surface pore size distributions with distinct similarities for the membranes cast from salt-containing solutions (Fig. 6). The PX0 membrane showed no evidence of surface pores from the SEM images analysed. Normal distribution fits of the data show that pore size distribution is consistent for PX10, PX20, PX40 and PX60, with peaks around the 2 µm diameter range (Fig. 6) and the different membranes all overlap in their pore sizes (Table 2).

**Table 2:** Mean surface pore size on skin layer of flat sheet membranes. Data shown represents mean ± SD (PX10 n = 93; PX20 n = 144; PX40 n = 216; PX60 n = 341).

**Flat sheet membrane formulation**	**Mean surface pore diameter (µm)**
PX0	Not porous
PX10	1.6 ± 0.6
PX20	2.2 ± 0.7
PX40	2.3 ± 0.7
PX60	2.0 ± 0.9

The slightly smaller pore size measured in the PX10 membranes could be due to the lower proportion of salt in the casting solution, and thus a smaller outflow of saline solution escaping through the membrane skin. This would be consistent with a liquid-liquid de-mixing hypothesis, as opposed to the salt templating hypothesis, but further investigation would be required to confirm this possibility. This is considered further in the discussion.

**Fig. 7:**
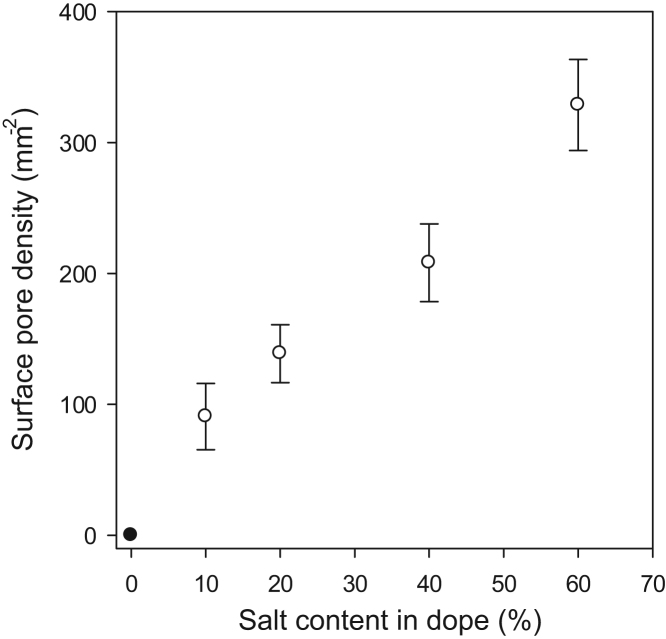
Surface pore density on flat sheet membranes, determined by SEM image analysis. Data represents mean ± SD (n = 8).

Surface pore density was calculated by SEM image analysis (Fig. 7). The number of surface pores was clearly seen to increase with salt proportion. As the pore size remains consistent across the different membranes while pore frequency increases, the use of microcrystalline sodium chloride as a pore-forming agent at different concentrations provides a simple method to tailor membrane porosity while decoupling porosity from pore size.

### 3.2 Hollow fibre characterisation

#### 3.2.1 Morphological analysis

**Fig. 8:**
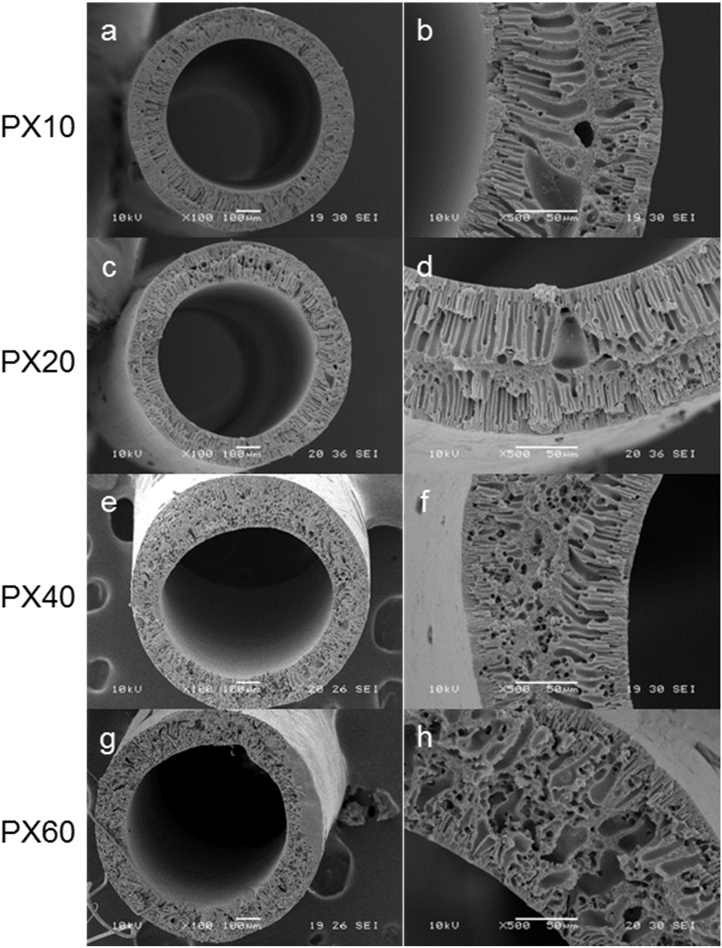
SEM micrographs of hollow fibre cross-sections. Typical structures of (a, b) PX10; (c, d) PX20; (e, f) PX40; (g, h) PX60. Left hand column scale bars represent 100 µm; right hand column scale bars represent 50 µm.

**Fig. 9:**
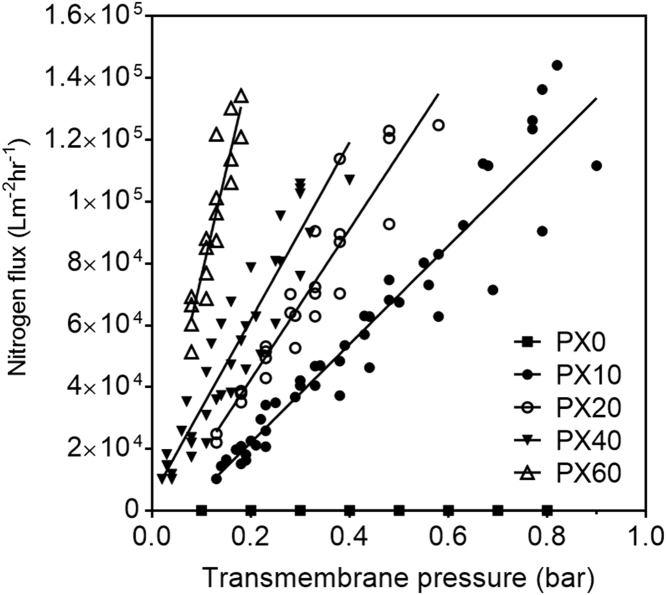
Flux of nitrogen through hollow fibres measured at a range of transmembrane pressures (n = 4). PX0 fibres were not gas permeable and ruptured at approximately 1 bar.

The morphologies of the PX10, PX20, PX40 and PX60 hollow fibres were examined using SEM (Fig. 8). The lumens are central in the fibres, and as the polymer is in contact with the non-solvent on both the inner and outer surfaces during production, the macrovoid/skin structure observed for the flat membranes (Fig. 5) is mirrored across the fibre with a skin layer on both surfaces. Pore connectivity is observed throughout the cross-section, and increased salt concentration appears to result in a less ordered structure overall. This may be due to the process of formation being more stochastic in nature due to salt dissolution, as well as the resultant saline outflow from the polymer. While fibres can be made using PX0 they are not analysed here due to their lack of porosity, which makes them unsuitable for use as membranes in a permeation-based system (this is illustrated further in Fig. 9).

Dimensions of the fibres displayed consistent uniformity (Table 3). However, caution must be exercised as small differences in lumen diameter result in sizeable differences in lumen surface area. This parameter is critical to the measurement of flux in pressure-driven membrane filtration, and for modelling properties such as solute diffusion through the structure.

**Table 3:** Hollow fibre dimensions as deduced from SEM micrograph image analysis. Data shown represents mean ± SD (n = 16).

**Fibre formulation**	**Fibre outer diameter (µm)**	**Wall thickness (µm)**	**Lumen diameter (µm)**	**Lumen surface area (mm^2^ m^−1^)**
PX10	914 ± 4.5	141 ± 8.5	632 ± 4.0	1984 ± 13
PX20	912 ± 6.2	139 ± 9.2	633 ± 3.0	1989 ± 9.3
PX40	919 ± 24	133 ± 17	654 ± 30	2053 ± 95
PX60	942 ± 7.5	142 ± 14	657 ± 6.6	2065 ± 21

#### 3.2.2 Permeability and mean pore size

Nitrogen gas permeation of the hollow fibres was measured in order to quantify differences in fibre permeability. The flux of nitrogen gas through the fibres was measured at various pressures (Fig. 9). The fibres produced from the different casting solutions gave distinguishable responses to this test and were tested to failure. The PX10 membrane was able to withstand transmembrane pressures of 0.9 bar. The maximum tolerated pressure decreased with increasing salt content, in line with the mechanical integrity data obtained from the flat sheet membranes (Section 3.1.2).

Linear regression analysis of the best-fit lines through each data set in Fig. 9 gives a measure of overall fibre specific permeability (Table 4). In line with the expectation that increased salt content in the casting solution leads to a more porous membrane, the permeability of the fibres increases in line with salt content. It is possible that the value recorded for PX40 is an underestimation of the true permeability, as a result of more variance in the fibre dimensions (as revealed by the higher standard deviations of the PX40 measurements in Table 3). The value for PX40, at 2.9 × 10^5^ Lm^−2^ h^−1^ bar^−1^, might be expected to be higher, midway between the values for PX20 and PX60. The mean pore size values show that while there is a direct correlation between increased salt content and mean pore size, the dimensions are considerably smaller than those measured in the top layer of the skin. This suggests that the mean pore size results from a combination of the thermodynamic interactions of the ternary system and the dissolution of the salt crystals. This is further supported by the fact that the mean pore sizes are four times smaller than the salt crystal size.

**Table 4:** Nitrogen permeability as determined from regression analyses of data in Fig. 9. Data shown represents the linear fit coefficient ± SE (n = 4). As PX0 fibres were not gas permeable they are excluded here.

**Fibre formulation**	**Nitrogen permeability (×10^5^ Lm^-2^ h^-1^ bar^-1^)**	**Mean pore size (µm)**
PX10	1.6 ± 0.1	0.26
PX20	2.5 ± 0.2	0.06
PX40	2.9 ± 0.2	1.01
PX60	7.0 ± 0.8	1.79

**Fig. 10:**
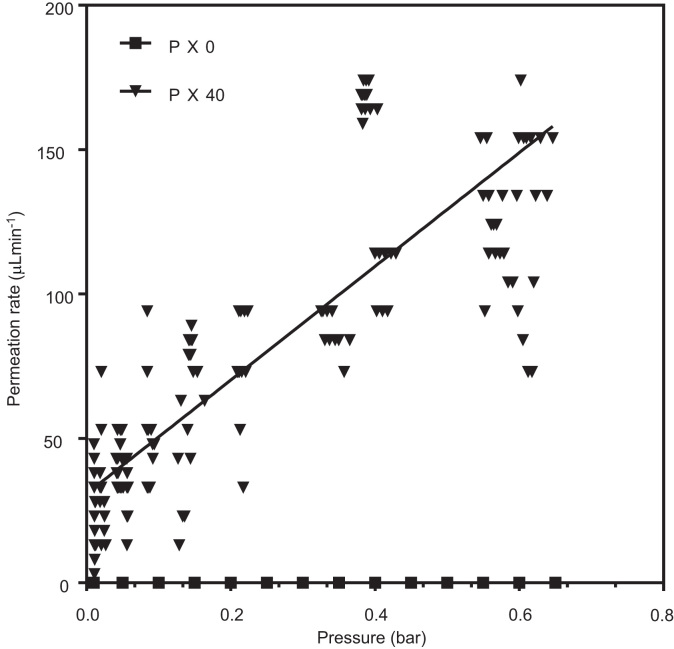
Permeation of water through a bundle of three PX hollow fibres measured at a range of transmembrane pressures (n = 3).

Based on the permeation data and physical properties of the membranes, PX40 was identified as the most suitable hollow fibre material for cell culture applications. To confirm the permeability of the membranes in a liquid system, water permeation of the fibres was carried out (Fig. 10). The data shows that PX40 membranes were water permeable even at low pressures. PX0 was again found to be non-permeable.

### 3.3 Cell response to PX membranes

#### 3.3.1 Cell viability

**Fig. 11:**
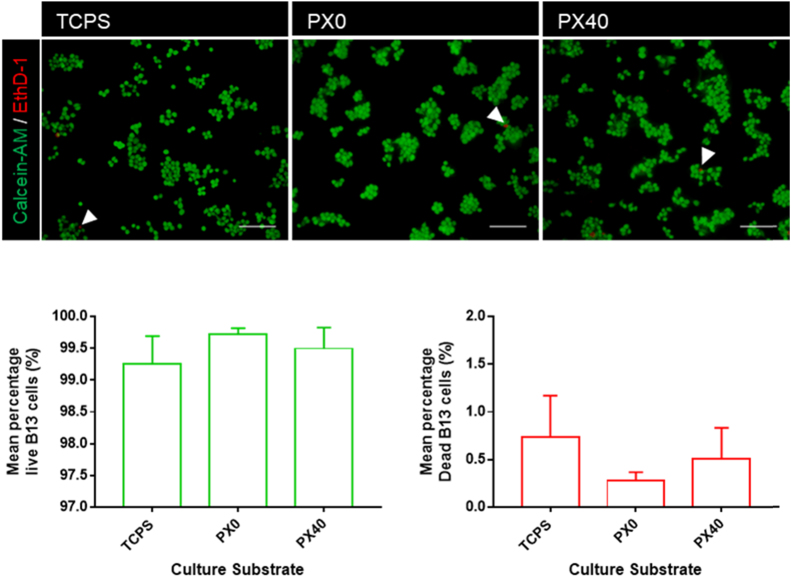
Viability of B13 cells seeded on TCPS, PX0 and PX40 membranes after 48 h of culture. Live cells are stained green; dead cells are stained red. White arrowheads indicate dead cell staining. Date shown represents mean + SE; N = 3. No significance was observed. Scale bar represents 100 µm. (For interpretation of the references to color in this figure legend, the reader is referred to the web version of this article.)

Having identified PX40 membranes as the most suitable for cell culture applications, the cell compatibility of the membranes was examined using pancreatic B13 cells. Standard tissue culture polystyrene (TCPS) plates and PX0 membranes were included as controls. Pancreatic B13 cells seeded onto TCPS, PX0 and PX40 membranes were stained for viability and representative images are shown in Fig. 11. All membrane surfaces showed the presence of attached cells with very high levels of viability (> 99%), indicated by the presence of calcein AM staining and the absence of ethidium homodimer-1 staining. No significant differences in viability between the B13 cells on the different culture substrates was observed. Cells do not adhere to untreated PX or polystyrene surfaces due to their hydrophobicity (Fig. 4), so only oxygen plasma treated membranes can be examined for cell viability and transdifferentiation.

#### 3.3.2 B13 cell transdifferentiation to hepatocyte-like cells on PX membranes

**Fig. 12:**
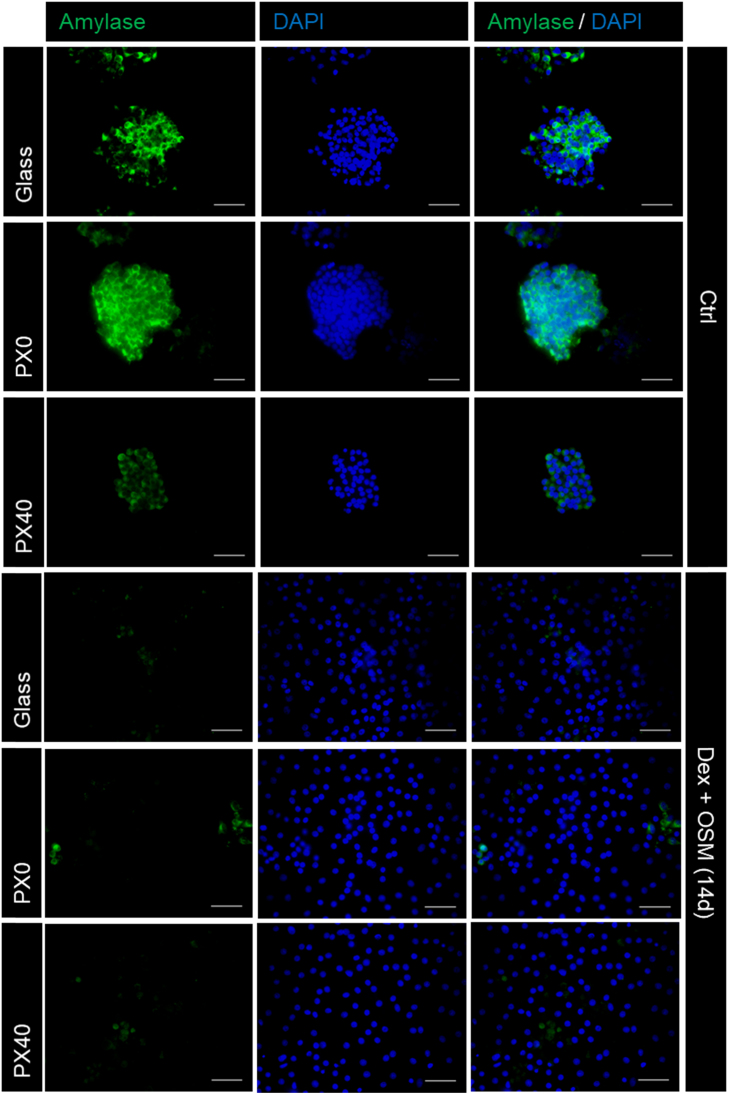
Expression of the pancreatic marker amylase in B13 cells cultured on glass, PX0 and PX40 for 4 days (control conditions) or 14 days (Dex and OSM). Cells were stained for the pancreatic marker amylase (green) and nuclei were stained with DAPI (blue). Dex and OSM treated cells on all substrates showed morphologically flattened cells with weak to absent amylase expression. Scale bar represents 50 µm. (For interpretation of the references to color in this figure legend, the reader is referred to the web version of this article.)

**Fig. 13:**
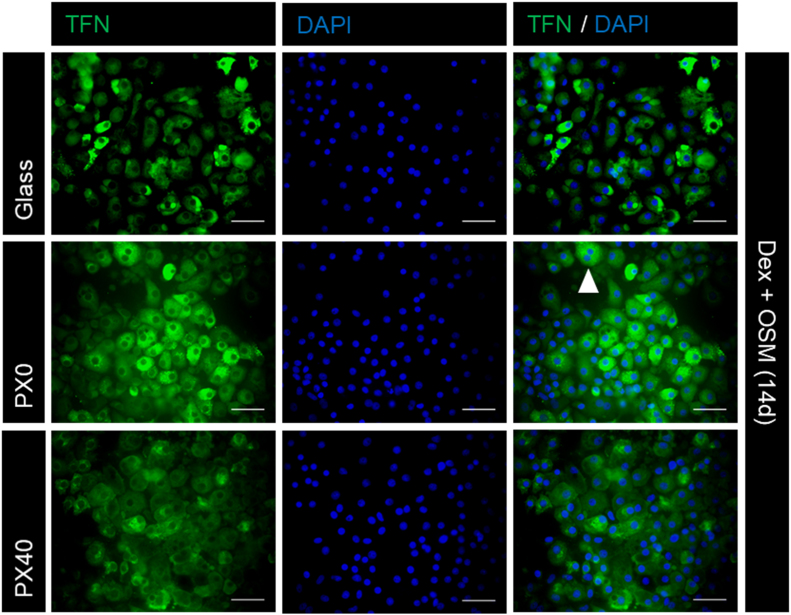
Expression of TFN in Dex and OSM treated B13 cells cultured on glass, PX0 and PX40 substrates for 14 days. Cells were stained for the hepatic transporter protein TFN (green) and nuclei were stained with DAPI (blue). The white arrowhead indicates a bi-nucleated cell. Dex and OSM treated cells had an enlarged, flattened morphology and demonstrated expression of TFN on all substrates. Scale bar represents 50 µm. (For interpretation of the references to color in this figure legend, the reader is referred to the web version of this article.)

**Fig. 14:**
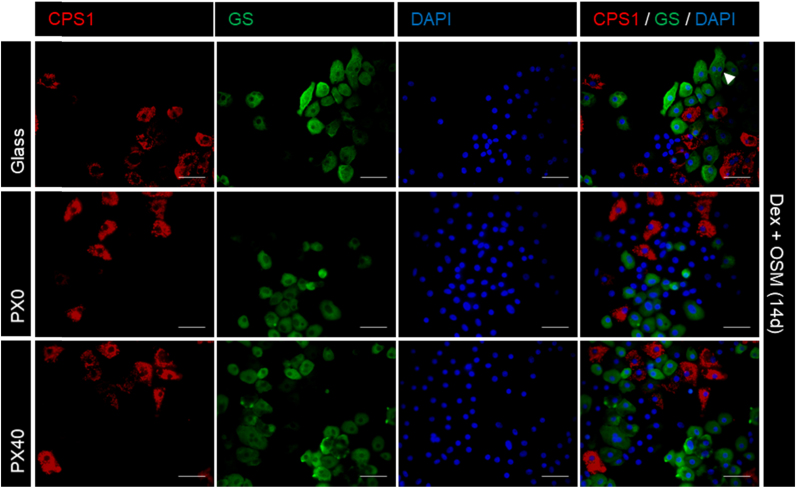
Expression of GS and CPS-1 in Dex and OSM-treated B13 cells cultured on glass, PX0 and PX40 for 14 days. Cells were stained for hepatic enzymes CPS-1 (red) and GS (green). Nuclei were stained with DAPI (blue). The white arrowhead indicates a bi-nucleated cell. Dex and OSM treated cells had an enlarged, flattened morphology and demonstrated expression of GS and CPS-1 on all substrates. Scale bar represents 50 µm. (For interpretation of the references to color in this figure legend, the reader is referred to the web version of this article.)

The ability of the B13 cell line to transdifferentiate to HLCs on PX membranes was assessed, using glass coverslips as a control substrate. Immunofluorescent staining showed that untreated B13 cells maintained expression of amylase, a pancreatic marker, but that B13 cells treated with Dex and OSM lost amylase expression (Fig. 12) and gained expression of the hepatic marker transferrin (TFN) (Fig. 13), the periportal hepatic marker carbamoylphosphate synthetase (CPS-1) and the perivenous hepatic marker glutamine synthetase (GS) on all culture substrates (Fig. 14). Treated cells also displayed an enlarged, flattened morphology, and populations of mono-nucleate and bi-nucleate cells, indicative of HLCs (Fig. 13). This is consistent with previous observations of treated B13 cells on glass coverslips [19], [29], [30]. No TFN, GS or CPS-1 expression was observed in the control untreated samples (Supplementary data: Figs. S2 and S3).

**Fig. 15:**
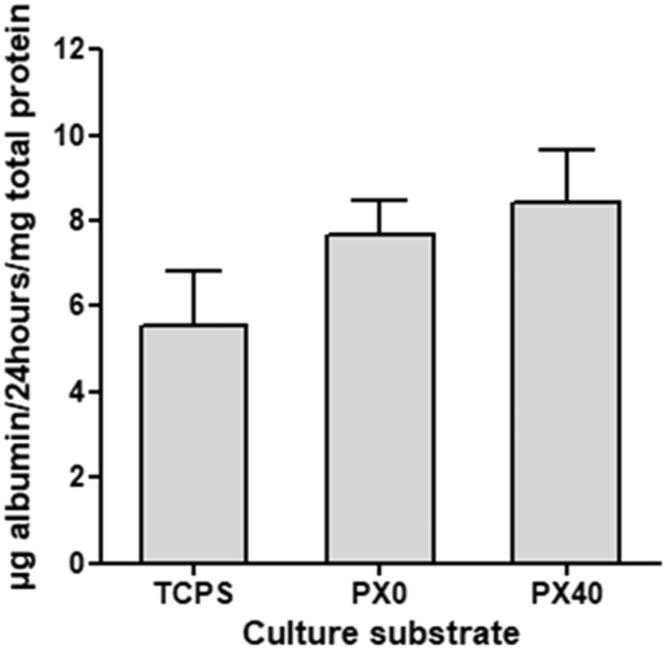
Relative serum albumin secreted over 24 h from B13 cells treated with Dex and OSM on TCPS, PX0 and PX40 for 14 days. Data shown represents mean + SE; N = 3. No significance was observed.

Dex and OSM treated B13 cells were also shown to secrete albumin on both PX membranes and TCPS (Fig. 15). Levels of secreted albumin were highest on PX40 membranes, followed by PX0. However, the differences between the substrates were not significantly different.

## 4 Discussion

### 4.1 Fibre fabrication and characterisation

This study has demonstrated that it is possible to tailor the porosity of polystyrene membranes, both as flat sheets and hollow fibres, by varying the concentration of microcrystalline sodium chloride in the polymer casting solution. The varied mechanical properties of the membranes can be attributed to the reduced material-to-pore ratio (lower bulk density) with increased salt content. The pores within the membranes show similar arrangements to those in other phase inversion-cast membranes described elsewhere [3], [31], [32].

The structural differences between PX0 membranes and those produced from the salt-containing solutions could either be a result of undissolved salt crystals bridging layers (and the film surface) during the precipitation process (the templating hypothesis), or of salt dissolution producing a saline solution which de-mixes in the membrane at a different rate to pure water (thermodynamic liquid-liquid de-mixing hypothesis). It is uncertain which of, or in what ratio, these mechanisms drive the pore structure formation, though a contribution by one or both leads to a disruption of the more organised, stratified layers identified in the salt-free membranes.

In the templating hypothesis, pores are created as a result of polymer precipitation around porogen ‘templates’ (in this case, microcrystalline sodium chloride). It follows that the size of the pores should therefore be related to the size of the porogen. However, the surface pores observed in Fig. 5 are much smaller than the ~ 5 µm microcrystalline sodium chloride. This could be due to salt crystals merely protruding into, or out of, the polymer surface, rather than occupying it completely and thus leading to smaller pores. It is also possible that the salt begins to dissolve before the polystyrene is fully coagulated, and therefore the observed pores are smaller than the measured size of the dry salt crystals.

On the other hand, the difference in porosity between the different membranes may be due to the different de-mixing mechanisms of water-NMP compared to saline-NMP. The energy change caused by the dissolution of sodium chloride in water is also likely to have an effect on the de-mixing of the solvents. For a clearer understanding of this system it would be necessary to investigate the specific thermodynamics of this process. While determining the absolute molar ratios of the respective solvent components within a dynamic system is difficult and cannot be easily measured, it may be possible to elucidate a trend between varied concentrations of the system components.

Within the membrane sub-layers, the observed macrovoids are much larger than the microcrystalline sodium chloride porogen, and the templating hypothesis is unlikely to be dominating the structure formation here. The macrovoids also increase in size with increasing concentration of salt in the casting solution.

The membranes themselves are formed by phase inversion, which has previously been suggested to be caused by one of two mechanisms:

i. instantaneous liquid-liquid de-mixing in the immersed dissolved polymer;
ii. delayed liquid-liquid de-mixing in the solubilised polymer whereby film properties are not affected by phase separation [33].

The transition between these thermodynamically-dictated states is a factor in the occurrence of macrovoids [33]. One factor contributing to the enhancement of macrovoids is the specific pairing of solvent and non-solvent, with high mixing affinity contributing to greater macrovoid formation [34], [35]. This can also be achieved with the inclusion of solvent in the coagulation bath. In short, shifting the ternary system to a state of instantaneous de-mixing contributes to macrovoid formation.

While macrovoids in membrane structures can sometimes be seen as unfavourable, as they may result in mechanical weaknesses in high pressure operating systems, for the *in vitro* liver model applications described here the membranes would be kept in low shear, low pressure environments [3]. In these environments, an open, macrovoid structure is desirable to maximise perfusion across the membrane.

### 4.2 Biocompatibility and cell response

Viability staining of B13 cells on TCPS, PX0 and PX40 showed attachment to all biomaterial surfaces after 48 h, demonstrating excellent viability and very low numbers of dead cells (Fig. 11). Oxygen plasma treatment of the polystyrene membranes significantly decreased the water contact angle measurements, indicating an increase in hydrophilicity and therefore allowing good cell attachment (Fig. 4). Treatment of PX membranes with the antibiotic-antimycotic solution previously recommended for sterilising PLGA membranes prior to culture is a suitable treatment for sterilisation as no infections were detected over the 14 day culture period [24].

Treatment of the B13 cells with Dex and OSM on PX membranes over 14 days induced transdifferentiation towards a hepatic phenotype. There was a distinct loss of the pancreatic phenotype shown through loss of expression of the pancreatic marker amylase, replicating the response observed on glass. Furthermore, expression of the hepatic markers TFN, CPS-1 and GS were found to be induced in the Dex and OSM treated cultures, and not the untreated samples on all culture substrates. This is a significant observation as it shows that the loss of pancreatic phenotype coincides with induction of hepatic markers, as previously described in the literature [19], [29]; and secondly, the culturing of B13 cells on PX membranes in complete B13 culture medium alone does not induce transdifferentiation of B13 cells to HLCs.

Transdifferentiated HLCs cultured on PX membranes were also able to demonstrate functional capability by secreting serum albumin into the culture medium. The amount secreted was slightly higher from cells cultured on PX membranes than on TCPS controls, but this difference was not significant.

Overall it was shown that PX membranes supported B13 attachment, viability and function at levels equivalent or greater than glass and TCPS controls, suggesting that these materials are ideally suited for use in cell culture applications – specifically for the generation of bioartificial liver devices based on membrane bioreactors. Indeed, PX40 hollow fibres have already been applied in such a system [1]. The fibres could be of interest for incorporation into commercial HF systems such as FiberCell, Terumo or Cellab, and in theory, any HF application where cells are currently cultured on standard tissue culture polystyrene.

## 5 Conclusions

This work describes for the first time the use of microcrystalline sodium chloride as a porogen in the development of a porous polystyrene membrane. Porous membrane formation was achieved under mild and economic conditions, resulting in a cost-efficient process. Varying the concentration of the porogen in the casting solution allowed control over the final membrane porosity, with a higher concentration resulting in a more porous membrane. However, average pore size was not affected by the change in porogen concentration, nor were the dimensions of the resultant membranes. Oxygen plasma treated polystyrene flat sheet membranes have been shown to support cell attachment and viability comparably to TCPS. The ability of the B13 cell line to transdifferentiate to HLCs when cultured on the developed PX membranes has also been established.

Further work is necessary to investigate B13 cell biological function and drug metabolism behaviour on PX hollow fibres, but the work presented here suggests the combination of B13 cells with PX membranes could be a valuable tool in the development of improved bioartificial liver models and devices.
