# Supplementary material for: Next generation in vitro liver model design: Combining a permeable polystyrene membrane with a transdifferentiated cell line
Source: J Memb Sci. 2018 Nov 1;565:425–38. doi: 10.1016/j.memsci.2018.07.063 (PMC6148409; doi:10.1016/j.memsci.2018.07.063)
Supplement: Supplementary file 1 — Supplementary material [file mmc1.docx]

# Supplementary data

## S1.1 Salt crystal characterisation

Different methods of preparing sodium chloride porogens were compared by image analysis (Figure S1). Microcrystalline sodium chloride prepared by the preparation of a frozen, saturated sodium chloride solution dispersed in cold ethanol appeared homogenous (Figure S1a). In Figure S1b, S1c and S1d, the microcrystalline salt structure is compared to ground salt and unprocessed salt. Though grinding is a possible alternative method for the production of fine salt crystals, the product is non-homogenous in both size and shape.


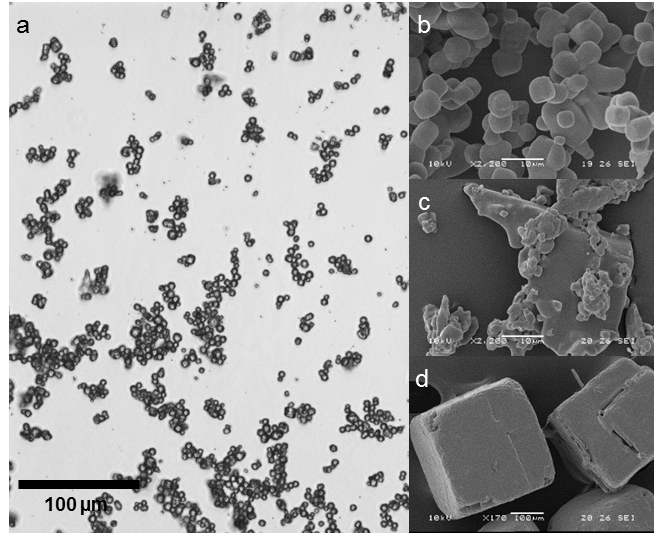


Figure S1: Micrographs of sodium chloride crystals. (a) Light micrograph of microcrystalline sodium chloride. SEM micrographs of (b) microcrystalline sodium chloride, (c) ground sodium chloride crystals, and (d) unprocessed sodium chloride. Scale bars in (a) and (d) represent 100 μm; scale bars in (b) and (c) represent 10 μm.

## S1.2 Immunofluorescent staining controls

Control cultures of B13 cells cultured on glass, PX0 and PX40, untreated with Dex and OSM, did not develop markers typical of hepatocytes or HLCs, unlike their treated counterparts (Figure S2& S3).


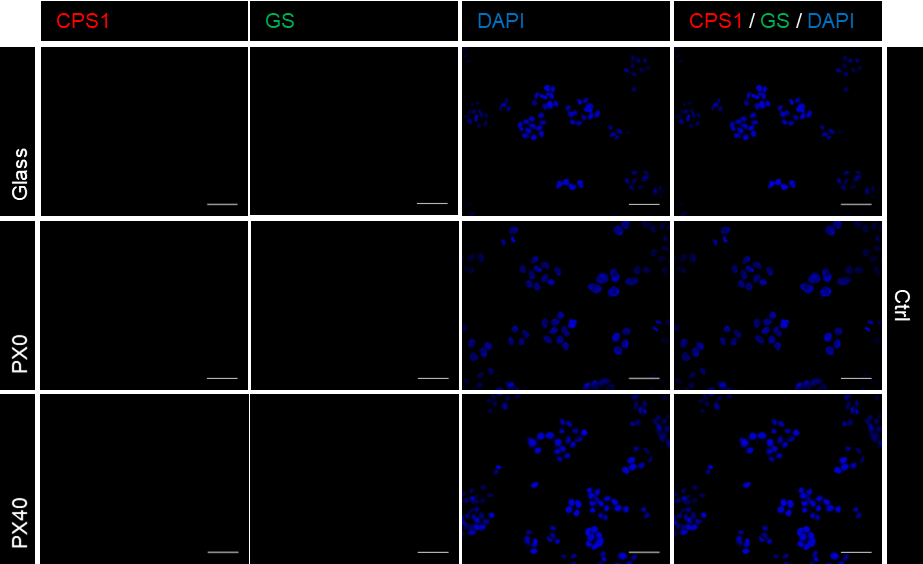


Figure S2: Expression of the ammonia detoxifying enzymes GS and CPS-1 in B13 cells cultured on different culture substrates for 4 days. Cells were stained CPS-1 (red) and GS (green). Nuclei were stained with DAPI (blue). Scale bars represent 50 μm.


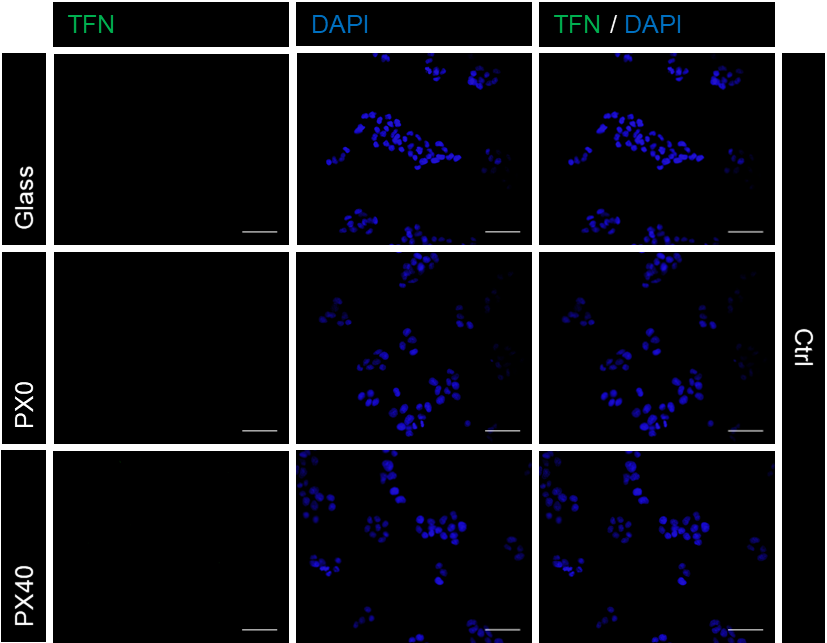


Figure S3: Expression of the hepatic transporter protein TFN in B13 cells cultured on different culture substrates for 4 days. Cells were stained for the hepatic transporter protein TFN (green) and nuclei stained with DAPI (blue). Scale bar represents 50 μm.
